# A Japanese girl with mild xeroderma pigmentosum group D neurological disease diagnosed using whole-exome sequencing

**DOI:** 10.1038/s41439-020-0109-z

**Published:** 2020-08-07

**Authors:** Takayuki Yokoi, Yumi Enomoto, Tomoko Uehara, Kenjiro Kosaki, Kenji Kurosawa

**Affiliations:** 1 Department of Pediatrics, The Jikei University School of Medicine, Tokyo, Japan; 2 Division of Medical Genetics, Kanagawa Children’s Medical Center, Yokohama, Japan; 3 Clinical Research Institute, Kanagawa Children’s Medical Center, Yokohama, Japan; 4 Center for Medical Genetics, Keio University School of Medicine, Tokyo, Japan

**Keywords:** Paediatric neurological disorders, Medical genetics

## Abstract

We report a Japanese girl with mild xeroderma pigmentosum group D neurological disease. She had short stature, cataracts, intellectual disability, and mild skin symptoms. However, she was not clinically diagnosed. Using whole-exome sequencing, we identified compound heterozygous pathogenic variants in *ERCC2*. In the future, the patient may develop skin cancer and her neurological symptoms may progress. Early genetic testing is necessary to clarify the cause of symptoms in undiagnosed patients.

Xeroderma pigmentosum (XP) is an autosomal recessive disease characterized by skin symptoms, including photosensitivity and skin cancer. Sometimes, patients with XP also have neurological symptoms. There are a total of eight XP groups, including seven genetic complementation groups and variants^1,2^. Neurological symptoms are associated with groups A, B, D (XPD: OMIM#278730), and G. Approximately 50% of all Japanese patients with XP are assigned to group A, 25% are assigned to the XP variant group, and XPD is rarely observed^3^. In Japan, more than 90% of XPD cases are classified as only having skin symptoms^3^. The gene responsible for XPD is *ERCC2*, which encodes an adenosine triphosphate (ATP)-dependent DNA helicase^4^. Here, we present a Japanese girl with mild XPD neurological disease characterized by short stature, cataracts, intellectual disability, and mild skin symptoms. Using whole-exome sequencing, we identified that she had compound heterozygous pathogenic variants in *ERCC2*.

The proband is a 15-year-old Japanese girl. Intrauterine growth retardation was noted during the course of pregnancy. She was born at 36 weeks and 4 days gestation without asphyxia to healthy, nonconsanguineous Japanese parents with a birth weight of 2045 g (−1.6 S.D.) and a length of 44.6 cm (−1.8 S.D.). No developmental delay was noted in infancy. The patient was diagnosed with cataracts and underwent bilateral cataract surgery at the age of nine. Her short stature was identified in early childhood, and she presented in another division of our hospital at the age of 10. Her height was three standard deviations below the norm at the time of initial presentation. She had no growth hormone deficiency or other endocrinological causes of short stature. Her height has consistently been ~3 standard deviations below the norm since presenting at our hospital. In the upper grades of elementary school, learning became more difficult. The Wechsler Intelligence Scale for Children-IV was conducted when she was 11 years of age and her IQ was 55. She entered a support school at the age of 12. To date, there has been no apparent regression. In general, she was not very good at exercising. She exhibited no muscle atrophy, hypotonia, or hypertonia. There were no other neurological symptoms or findings. At the age of 12, she was referred to our division for investigation of the cause of her multiple congenital anomalies and intellectual disability. There were no obvious abnormal facial findings (Fig. 1a). No other external physical deformities were observed. There was no hearing loss, and no skin symptoms were apparent. Brain MRI results showed no obvious abnormal findings. Muscle-related enzyme levels were normal. Myotonic dystrophy was ruled out through *DMPK* genetic testing. She had a normal female karyotype. Written informed consent was obtained from the parents of the patients in accordance with the Kanagawa Children’s Medical Center Review Board and Ethics Committee. Whole-exome sequencing was performed and revealed compound heterozygous pathogenic mutations in *ERCC2*, c.1445_1447delCCA: p. Thr482del and c.1003C>G: pArg335Gly, and we confirmed that they were inherited from the father and mother, respectively (Fig. 1b). The former mutation had already been reported^5^. The latter variant was estimated as likely pathogenic in the guidelines for the interpretation of sequence variants of American College of Medical Genetics^6^: 2 moderate (PM3: Absent from controls (or at extremely low frequency if recessive) in Exome Sequencing Project (absent), 1000 Genomes (absent) or ExAC (1.88e-5), and PM3: for recessive disorders, detected in trans with a pathogenic variant) and ≥2 supporting (PP3: Multiple lines of computational evidence support a deleterious effect of the gene or gene product (score of SIFT: 0 (deleterious), score of PolyPhen-2: 1 (probably damaging), score of MutationTaster: 1 (disease causing), and Combined Annotation Dependent Depletion score: 29.8 (most deleterious)) and PP4: Patient’s phenotype or family history is highly specific for a disease with a single genetic etiology)^6^. After genetic diagnosis, it was revealed that mild photosensitivity was observed in early infancy. The parents were concerned about the invasiveness of skin testing and did not consent to skin examinations, such as ultraviolet sensitivity and DNA repair tests.

**Fig. 1:**
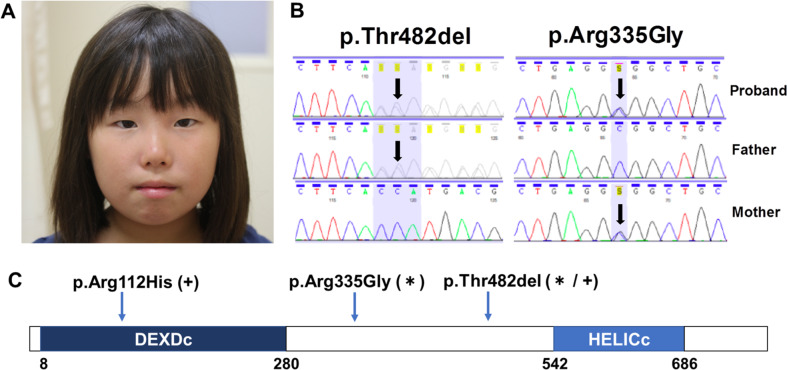
Clinical features and *ERCC2* pathogenic variants of this patient. **a** Photograph of the patient at 14 years of age, which was permitted to be presented in any journal by her parents. The patient had no abnormalities, including skin abnormalities. **b** The variants in ERCC2 identified in the patient by targeted sequencing. Sanger sequencing demonstrated inheritance from parents. **c** The *ERCC2* structure and loci of variants of our patient (indicated by asterisk) and reported case (indicated by plus)^5^. DEXDc DEAD-like helicase superfamily, HELICc helicase superfamily c-terminal domain.

The proband was difficult to clinically classify. We determined that XPD neurological disease was the most applicable diagnosis. This diagnosis was based on the proband’s marked short stature, cataracts in her youth, mild intellectual disability, and mild photosensitivity. There was no obvious progression of neurological symptoms. Cockayne syndrome is associated with photosensitivity, a characteristic face, atrophy of subcutaneous fat, short stature, and marked nutritional disorders. XPD with Cockayne syndrome is rarely observed^7,8^. The facial characteristic associated with Cockayne syndrome were not seen in the proband. Although XP may be associated with trichothiodystrophy, no abnormal hair or ichthyosis was observed in the proband. In fact, these clinical classifications are ambiguous from previous reports^7^. Although skin symptoms were mild in the proband, skin cancer may develop at a comparatively early age. Furthermore, neurological symptoms may progress with age. Therefore, early definitive diagnosis is desirable for making concrete and strict observations of relevant symptoms. One of the pathogenic *ERCC2* variants identified in the proband was already reported, and the other was novel. The p.Thr482del variant was previously reported in a patient diagnosed with XPD^5^. That patient’s symptoms were also complicated with trichothiodystrophy and severe neurological symptoms (Table 1)^9^. The other variant in that case, p.Arg112His, was located in a DEAD-like helicase superfamily domain (Fig. 1c). In this study, the pArg335Gly mutation was located in an inactive domain site in the proband. Together, these data may indicate that p.Arg112His influences the presence and severity of trichothiodystrophy. Presently, there are no genotype–phenotype correlations in XPD (with or without neurotic symptoms), XP-CS, or trichothiodystrophy. The association between pathogenic variants, their combinations, and phenotypes is complicated, and clarification of these relationships requires the continual assessment of additional cases.

**Table 1:** Comparison of clinical features of patients with XP, CS, trichothiodystrophy, and this patient^10^.

	CS	Trichothiodystrophy	XP neurological disease	Patient with reported case^9^	This proband
Photosensitivity	+	+	+	+	+
Increased freckling	−	+	+	−	−
Skin cancer	−	+	+	−	−
Dwarfism	+	+	±	+	+
Intellectual disability/developmental delay	+	+	+	+	+
Brain anomaly	+	+	−	NA	−
Skeletal abnormalities	+	±	−	+	−
Cataract	+	+	+	+	+
Sensorineural deafness	+	−	+	NA	−
Recurrent infections	+	+	−	−	−
Ichthyosis	−	+	−	−	−
Brittle hair	−	+	−	+	−
Other neurological symptoms	+	+	+	+	−

*XP* xeroderma pigmentosum, *CS* Cockayne syndrome, *NA* not available.

In conclusion, we present a patient with XPD neurological disease and pathogenic variants in *ERCC2*. It is relatively difficult to clinically identify or suspect this disease based on only mild intellectual disability, early cataracts, and short stature. Although this patient had multiple congenital anomalies and an intellectual disability that appeared nonprogressive, these deficits may become progressive or the patient may develop cancer. Therefore, it is necessary to actively implement comprehensive genetic testing to allow for early diagnosis and continual and accurate monitoring.

## Supplementary information

Supplementary Information

## Data Availability

The relevant data from this Data Report are hosted at the Human Genome Variation Database at 10.6084/m9.figshare.hgv.2876.
