# Supplementary material for: A Japanese girl with mild xeroderma pigmentosum group D neurological disease diagnosed using whole-exome sequencing
Source: Hum Genome Var. 2020 Aug 7;7:22. doi: 10.1038/s41439-020-0109-z (PMC7414221; doi:10.1038/s41439-020-0109-z)
Supplement: Supplementary file 1 — Supplementary Information [file 41439_2020_109_MOESM1_ESM.docx]

**SUPPORTIG INFORMATION**

Total genomic DNA was obtained from lymphocytes using QIAamp DNA Blood Mini Kit (Qiagen, Valencia, CA, USA), following the manufacturer’s instructions.

DNA libraries were enriched for whole exome sequences using SureSelect XT human ALL Exon Kit V6 (Agilent Technologies Inc., Santa Clara, CA, USA). Patient samples were sequenced by HiSeq 2500 (Illumina Inc., San Diego, CA, USA). Data were analyzed by Burrows–Wheeler alignment tool and the Genome Analysis Toolkit pipeline (Broad Institute, Cambridge, MA, USA), and visualized in the Integrative Genomics Viewer (IGV). Calling copy-number variation (CNV) was based on log–ratio analysis and read depth *z*-score of each exon. Mutations identified by targeted sequencing were confirmed by Sanger sequencing and demonstrated appropriate segregation with phenotype in the unaffected parents.

The databases used to validate variants were: Human Genetic Variation Database (the Japanese genetic variation consortium: a reference database of genetic variations in the Japanese population comprising 1,208 individuals [http://www.hgvd.genome.med.kyoto-u.ac.jp]), the 1000 Genomes project (https://www.internationalgenome.org), National Heart, Lung, and Blood Institute (NHLBI) grant opportunity exome sequencing project (ESP) (https://esp.gs.washington.edu/drupal/), The Human Gene Mutation Database (HGMD) professional (http://www.hgmd.cf.ac.uk/ac/index.php), Exome Aggregation Consortium (ExAC) (instead of ExAC, equivalent data is now available from the Genome Aggregation Database (https://gnomad.broadinstitute.org)), and in-house control Japanese genomic samples. Functional predictions of variants were performed using mainly SIFT (http://sift.jcvi.org/), Polyphen-2 (http://genetics.bwh.harvard.edu/pph2/), MutationTaster (http://neurocore.charite.de/MutationTaster/), and Combined Annotation Dependent Depletion (https://cadd.gs.washington.edu).
